# Expression of Thyroid Hormone Responsive SPOT 14 Gene Is Regulated by Estrogen in Chicken (*Gallus gallus*)

**DOI:** 10.1038/s41598-017-08452-6

**Published:** 2017-08-31

**Authors:** Junxiao Ren, Naiyi Xu, Hang Zheng, Weihua Tian, Hong Li, Zhuanjian Li, Yanbin Wang, Yadong Tian, Xiangtao Kang, Xiaojun Liu

**Affiliations:** 1 College of Animal Science and Veterinary Medicine, Henan Agricultural University, Zhengzhou, 450002 China; 2 Henan Innovative Engineering Research Center of Poultry Germplasm Resource, Henan Agricultural University, Zhengzhou, 450002 China; 3 International Joint Research Laboratory for Poultry Breeding of Henan, Henan Agricultural University, Zhengzhou, 450002 China

## Abstract

Thyroid hormone responsive spot 14 (THRSP) is a small nuclear protein that responds rapidly to thyroid hormone. It has been shown that THRSP is abundant in lipogenic tissues such as liver, fat and the mammary gland in mammals. The *THRSP* gene acts as a key lipogenic activator and can be activated by thyroid hormone triiodothyronine (T3), glucose, carbohydrate and insulin. Here we report that chicken THRSP is also abundant in lipogenic tissues including the liver and the abdominal fat, and its expression levels increased with sex maturation and reached the highest level at the peak of egg production. Structure analysis of the *THRSP* gene indicates that there is a conscious estrogen response element (ERE) located in the −2390 – −2402 range of the gene promoter region. Further studies by ChIP-qPCR proved that the ERα interacts with the putative ERE site. In addition, *THRSP* was significantly upregulated (*P* < 0.05) when chickens or chicken primary hepatocytes were treated with 17β-estradiol in both the *in vivo* and *in vitro* conditions. We therefore conclude that THRSP is directly regulated by estrogen and is involved in the estrogen regulation network in chicken.

## Introduction

Spot 14, also referred to as thyroid hormone responsive spot 14 (THRSP), was originally identified in rat liver^1^. Immunoprecipitation and tissue distribution studies demonstrated that THRSP is primarily located in the nucleus and abundant only in lipogenic tissues such as liver, fat and the mammary gland^2, 3^. The THRSP is a small nuclear protein that is rapidly upregulated by thyroid hormone triiodothyronine (T3) signals that induce lipogenesis^4^. In addition, glucose, carbohydrate, polyunsaturated fatty acids and insulin can also influence and regulate THRSP transcription^5^. It has been shown that THRSP, as an important transcription factor in hepatocytes, is a lipogenic activator that controls the expression of several lipogenic genes^6–8^. Hepatocytes transfected with a *THRSP* antisense oligonucleotide express decreased mRNA levels in enzymes, such as ATP-citrate lyase (*ACLY*), fatty acid synthase (*FASN*) and malic enzyme (*ME*), involved in the lipogenic pathway^8–10^. Furthermore, an increase in lipogenesis was observed in the *THRSP* knockout mouse and *THRSP* overexpression increased mRNA levels of *FASN* in bovine mammary epithelial cells^11^.

The chicken *THRSP* gene was first identified by microarray analysis as an expressed sequence tags (EST), and subsequently mapped to 1q41–44^12^. This chromosomal region in chickens also harbors quantitative trait loci (QTL) for skin and abdominal fatness^13^. Comparative sequence analysis among mammalian and chicken *THRSP* genes demonstrated a similar gene organization with 2 exons and an intron, but the chicken THRSP peptide had a low similarity (less than 30% of identities and 50% of positives) to the THRSP amino acid sequences of mammals and fishes^14, 15^. Furthermore, the chicken *THRSP* genes appear to be predominantly expressed in lipogenic tissues such as the liver and abdominal fat of feeding nestling broiler chickens, and the polymorphisms in the coding regions of the paralogous genes were associated with the abdominal fatness of chickens^12, 14^.

Nuclear hormone receptors are ligand-activated transcription factors that regulate gene expression by interacting with specific DNA sequences upstream of the target genes. Thyroid hormone is secreted by the thyroid gland and plays an important role in the hypothalamus-pituitary-thyroid axis. Thyroid hormones function through a group of nuclear receptors, namely the thyroid hormone receptors (TRs), which interact with the hormone receptor elements (TREs) upstream of target genes. As a thyroid hormone responsive protein, three positive TREs were identified upstream of the *THRSP* gene in mammalian systems^16–18^. Moreover, there are also carbohydrate response elements (ChoRE)^4^ and sterol response elements (SRE)^19, 20^ in the promoter of the *THRSP* gene. The estrogen receptors (ERs) are also nuclear hormone receptors, mediate the physiological actions of estrogen and regulate the transcription of target genes^21, 22^ by forming dimers with estrogen and binding to a canonical 13-bp ERE, GGTCANNNTGACC (N represents any of the nucleotides), a palindromic inverted repeat separated by any three nucleotides (NNN)^23, 24^. Estrogen is vitally important for sexual maturity and the development of the female reproductive system^25, 26^.

Our previous work on the transcriptome of the liver in chickens showed that the *THRSP* gene was one of the differently expressed genes, which increased by 6.24-fold in peak-laying hens (30 weeks old) compared with pre-laying hens (20 weeks old)^27^. It was generally suggested that the upregulated genes in the livers of laying hens were regulated by estrogen and involved in lipid metabolism given that in laying hens plasma estrogen was relative higher than in immature pullets for certain periods^28, 29^. Additionally, more lipoproteins, mainly very low density lipoproteins and vitellogenins, were synthesized from the hen’s liver and transported to rapidly growing oocytes^30^. Many differently expressed key lipid metabolism genes in our transcriptome were reported to be involved in the estrogen regulation network, such as *VTG2*, *ApoB* and very low density apolipoprotein II (*APOV1*). The *VTG2* is a member of a small gene family encoding the yolk precursor proteins, the expression of which is strictly dependent on estrogen^31–33^. The mature *APOV1* mRNA appears in chicken liver within a few hours after estrogen administration^34^. Moreover, the ERE sequence was originally identified by the conserved sequence of the estrogen-sensitive *Xenopus laevis VTG2* and the chicken *APOV1* genes^24^. *THRSP* has been postulated to be a lipogenic activator; however, whether the expression of *THRSP* is regulated by estrogen in the liver is unknown. Therefore, the objective of the present study was to investigate regulatory mechanisms of *THRSP* in the liver of laying hens.

## Materials and Methods

### Ethics Statement

The birds used in this study were Lushi green-shelled-egg chickens that were obtained from the Animal Center of Henan Agricultural University. The birds were raised in similar environmental conditions with food and water *ad libitum*. All animal experiments were approved by the Institutional Animal Care and Use Committee (IACUC) of Henan Agricultural University Zhengzhou, P.R. China (Permit Number: 11–0085) and performed in accordance with the protocol outlined in the “Guide for Care and Use of Laboratory Animals” (Henan Agricultural University). All efforts had been done to decrease animals suffering. All birds were euthanized with pentobarbital.

### Animals, treatment and sampling

To study the expression profiles of *THRSP*, 6 birds each at different development stages (10, 15, 20, 30 and 35 weeks old) were euthanized. A total of 11 tissues including the heart, liver, pectoral muscle, kidney, adrenal gland, spleen, abdominal fat, duodenum, glandular stomach, pancreas and lung were quickly removed, snap-frozen in liquid nitrogen and stored at −80 °C freezer until analysis.

To investigate the effect of 17β-estradiol on the expression of *THRSP*, a total of 70 birds at 10 weeks old were divided randomly into 7 groups, with 10 birds in each group. The first 3 groups of birds were injected intramuscularly (chest muscle) with 0.5 mg, 2.0 mg or 8.0 mg of 17β-estradiol (Sigma, St Louis, MO, USA) (dissolved in olive oil)/kg of body weight, respectively. The birds were euthanized 12 hours after treatment. Another 3 groups of birds were also injected with the same amounts of 17β-estradiol as it was done in the first 3 groups, but euthanized 24 hours after treatment. The last group of birds was served as control and injected with the same amount of solvent only. The liver and adipose tissues were collected and stored as mentioned above.

### Primary hepatocyte culture and treatment

The method of chicken primary hepatocyte isolation was the same as described previously^35, 36^. In brief, hepatocytes were isolated from 18-day chicken embryonic livers. A total of 1 × 10^6^ cells/well was seeded into 6-well plates. When the cells reached 90% confluence, cell culture medium was replaced by fresh serum-free DMEM medium containing 100 mg/mL streptomycin and 100 U/mL penicillin, and incubated for 6 hours. Cells were divided into four groups with triplicates, and were treated with 17β-estradiol dissolved in 0.1% ethanol at a final concentration of 25 nM, 50 nM or 100 nM, respectively. Cells treated with ethanol alone were used as controls. Cell culture medium was removed 24 hours after initial culture, washed twice with PBS, and followed by the addition of 1 mL of Trizol reagent (Takara, Kyoto, Japan) in each well. Cells were then collected by a cell scraper, and stored at −80 °C until use.

To further demonstrate that the expression of *THRSP* is regulated by estrogen, primary hepatocytes were treated with 100 nM of 17β-estradiol alone or in the presence of the ICI 182,780, an estrogen receptor antagonist at 1 μM. Cells treated with ethanol alone were used as controls. Six replicates were included in each group. Cells were collected 24 hours after treatment, and stored at −80 °C until use.

### RNA extraction and reverse transcription

Total RNA was extracted from different tissues and primary hepatocyte using Trizol reagent (Invitrogen, Carlsbad, CA, USA) according to the manufacturer’s instructions. Each of the RNA samples was treated with DNase I (Invitrogen #18068015) to remove trace amounts of genomic DNA according to the manufacturer’s instructions. The integrity of total RNA was confirmed by gel electrophoresis, and the RNA concentration was determined using a NanoDrop 2000 spectrophotometer (Thermo Scientific, Wilmington, DE, USA). The RNA samples with OD260/280 ratios above 1.8–2.0 were used for further study. Single-stranded cDNA was synthesized using random hexamer primers with the Thermo Scientific™ Revert Aid First Strand cDNA Synthesis kit (Thermo Scientific) following the manufacturer’s protocol. The cDNA was stored at −20 °C until use.

### Chromatin immunoprecipitation (ChIP)

Liver tissues collected from birds treated with 2.0 mg /kg of estrogen were used in the Chip assay. The ChIP assay was performed using a kit (Millipore, Billerica, MA, USA) according to the manufacturer’s protocol. Briefly, 100 mg chicken liver tissue were cross-linked with 1% formaldehyde for 15 min at 37 °C and stopped with 2.0 M glycine. Then the samples were re-suspended in SDS lysis buffer (50 mM Tris-HCl at pH 8.0, 1% SDS, 10 mM EDTA, and protease inhibitors). Chromatin were sheared by probe sonication (5 × 10 s pulses, 30% amplitude), to an average fragment size of 200–500 bp. The lysates were cleared by centrifugation and diluted with ChIP dilution buffer (16.7 mM Tris–HCl at pH 8.1, 0.01% SDS, 1.1% Triton X-100, 1.2 mM EDTA, 16.7 mM NaCl, and protease inhibitors). Cell lysates were incubated with an anti-ERα (0.2 mg/mL, Invitrogen Ltd, Paisley, UK Catalog #: MA5–13065) or an anti-IgG control antibodies (Cell Signaling, Boston, MA, USA) for 12 hours at 4 °C, and then captured with the Protein G-agarose (Santa Cruz, Dallas, TX, USA). The purified DNA (IP sample) was amplified by qPCR. Meanwhile, 20% of starting chromatin without chromatin immunoprecipitation was used as input to represent the unselected DNA content. Fold Enrichment Method was used to normalize the ChIP-qPCR data. Fold Enrichment = log_2_^−ΔΔCt^, ΔCt(THRSP) = Ct(IP)−Ct(Input) − log_2_^5^, ΔΔCt = ΔCt(THRSP) − ΔCt(IgG).

### Quantitative real-time PCR (qPCR)

The qPCR was performed using a LightCycler 96 system (Roche) by the SYBR Green method according to the method of Nolan^37^. Each individual sample for qPCR was run in triplicate. Chicken *β-actin* was used as an internal control. Each reaction contained 5 µl of SYBR Green PCR Master Mix (Takara), 3.5 µl of RNase-free water, 0.5 µl each of forward and reverse primers (10 μM) and 0.5 µl of extracted cDNA (1000 ng/μL). The amplification protocol was used as follows: denaturation at 95 °C for 5 minutes; followed by 40 amplification cycles at 95 °C for 30 seconds, annealing at 60 °C for 30 seconds and elongation at 72 °C for 20 seconds. After amplification, melting curve analysis was performed as follows: 95 °C for 10 seconds; annealing temperature of 65 °C for 20 seconds; continuous temperature gradient to 97 °C with 5 acquisitions/s. The expression levels were measured in terms of the cycle threshold (Ct) and then normalized to β-actin using the 2^−ΔΔCt^ method. The primers for qPCR were designed by the NCBI Primers-BLAST online programs (https://www.ncbi.nlm.nih.gov/tools/primer-blast/). The forward and reverse primers for qPCR and ChIP-qPCR are shown in Table 1.

**Table 1:** List of qPCR and ChIP-qPCR primers used in the study.

Primers name	Gene	Accession number	Primer sequence (5′-3′)	Product size
qPCR	*THRSP*	NM_213577.2	F: GTTCTGACCGACCTCACCAA	175
			R: GTGGGACTTGGCACAGGAAT	
	*APOV1*	NM_205483.2	F: CAATGAAACGGCTAGACTCA	108
			R: AACACCGACTTTTCTTCCAA	
	*THRα*	NM_205313.1	F: CATCTTCGACCTCGGCAAGT	143
			R: GGTACGTCTCCTGGCACTTC	
	*THRβ*	NM_205447.2	F: GTTCGCTATGACCCCGAGAG	91
			R: CTACGCCAAGACCCCCATTT	
	*AACS*	NM_001006184.1	F:ATGGTTCATTCAGCAGGGGG	108
			R: CCAGCCAGTCGTTGTGTAGT	
	*FASN*	NM_205155.2	F: TCTCTGCCATCTCCCGAACT	184
			R: GCCTTCCATTCTCTAACACA	
	*SCD*	NM_204890.1	F: CAAGTTCTCCGAGACGCATG	178
			R: GGGCTTGTAGTATCTCCGCT	
	*DIO1*	NM_001097614.1	F:GAGCATCAGGGTGCTCCTAC	182
			R: AAGTGGGACCCCAGTTTTCG	
	*DIO2*	NM_204114.3	F: GCTTCAGATCTTGCCGGTCT	130
			R: CTCCTCCAAGTTTGACCGC	
	*MC5R*	NM_001031015.1	F: TGTGCCTACTGTCAAGAGCA	211
			R: CTCCCAAGCATTAGACACGC	
	*ADHH1B*	NM_001305183.1	F: CTGAAATGACTGGCCAGGGT	116
			R: TCCCACAATCACGCTGACTC	
	*PIK3CA*	NM_001004410.1	F: ACACGTTCTTGTGCTGGCTA	177
			R: TAAGACAAAGGGCACACGCT	
	*β-actin*	NM_205518.1	F: GAGAGAAGATGACACAGATC	116
			R: GTCCATCACAATACCAGTGG	
ChIP-qPCR	*APOV1*	NM_205483.2	F: CCATTACCAAATCCGAACA	156
			R: CATCTGAGACACTGGCATTT	
	*THRSP*	NM_213577.2	F: AGGTGCCAACAAGTTCAGC	165
			R: TCTCCATCAGCCACCTCAG	

### Statistical analysis

Statistical analyses of the qPCR results were carried out using SPSS version 20.0. One-way ANOVA and repeated measures ANOVA were used for statistical analysis of relative expression levels, followed by Dunnett’s test. Graphics were drawn using Graphpad Prism 5 (Graphpad Software, San Diego, CA, USA), and differences were considered significant at *P* ≤ 0.05. Data were expressed as the mean ± SD.

## Results

### Expression patterns of *THRSP* in various tissues of chicken

**Figure 1:**
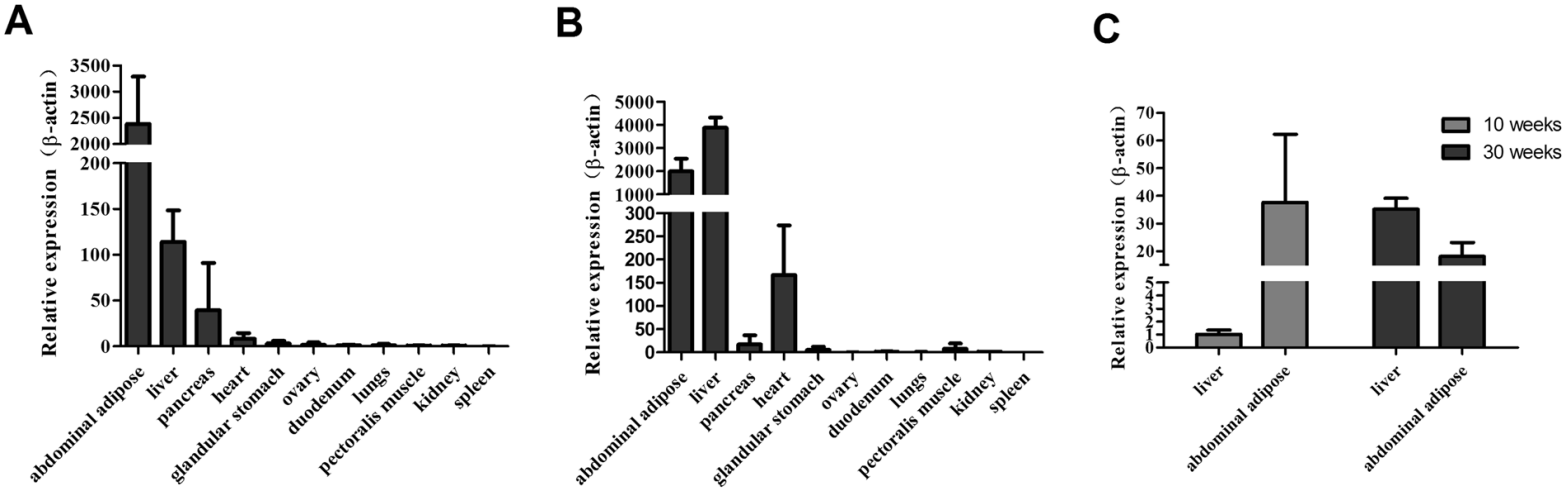
Tissue distribution of *THRSP* in different chicken tissues. (**A**) Tissue distribution of *THRSP* in 10 weeks old chicken tissues. (**B**) Tissue distribution of *THRSP* in 30 weeks old chicken tissues. (**C**) The mRNA levels of *THRSP* in liver and abdominal adipose of chickens at the age of 10 weeks old and 30 weeks old. The mRNA levels of the *THRSP* gene were normalized to the mRNA levels of *β-actin* gene. The data show as 1000 * 2^−ΔCt^ and represent the mean ± SD of 6 chickens (n = 6).

The expression patterns of *THRSP* in various tissues including the heart, kidney, liver, spleen, brain, muscle and white adipose tissue of 10-week and 30-week-old chickens were detected by qPCR. The results showed that expression of *THRSP* mRNA was mainly confined to the liver and adipose tissue, where its abundance was the highest, followed by the pancreas and heart (Fig. 1A and B). The level of THRSP expression was greater in abdominal fat than in the liver with 10-week-old chickens, but was less in the abdominal fat at 30 weeks. Interestingly, the results showed that an expected increase of 40-fold was detected in *THRSP* mRNA levels in the liver at 30 weeks compared with 10 weeks (Fig. 1C). However, a dramatic decrease of 2-fold was detected in *THRSP* mRNA levels in adipose tissue at 30 weeks compared with 10 weeks (Fig. 1C).

### Expression patterns of *THRSP* in the livers of chickens at different developmental stages

**Figure 2:**
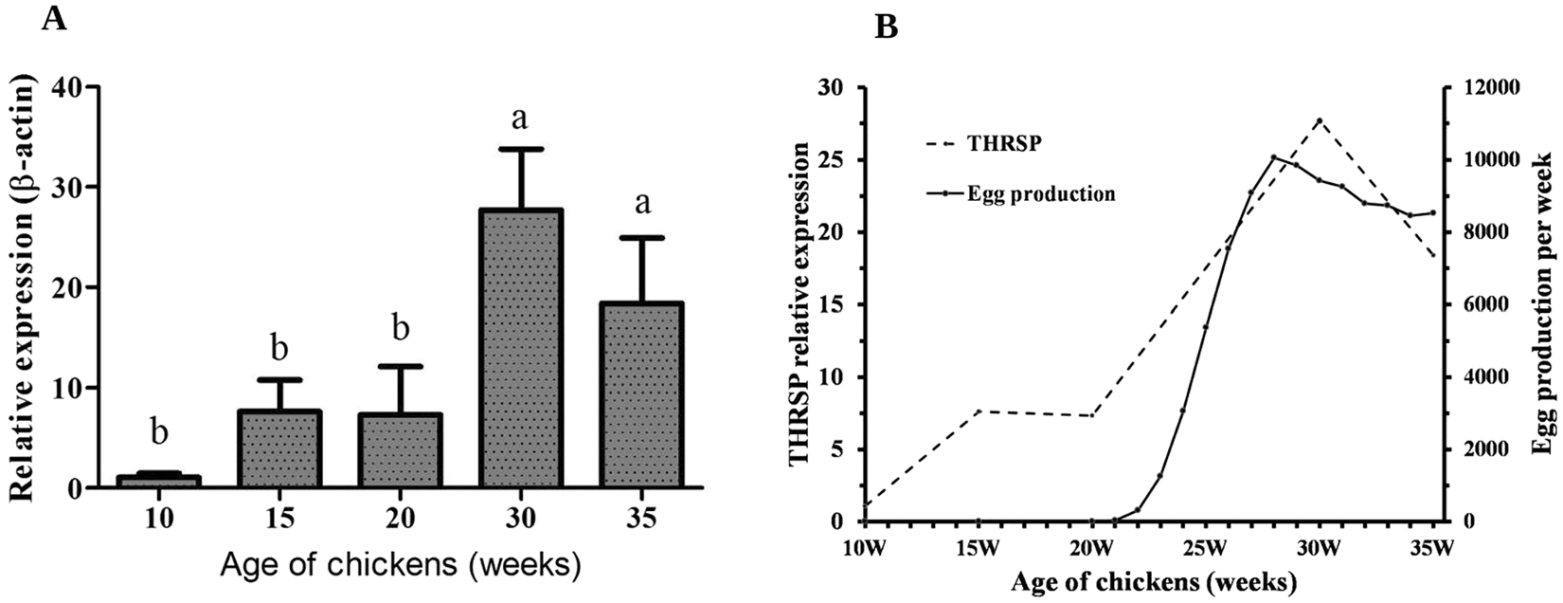
Expression patterns of *THRSP* in chicken livers and egg production curve analysis. (**A**) Expression patterns of *THRSP* in livers of chickens at different developmental stages. The mRNA levels of the *THRSP* gene were normalized to mRNA levels of β-actin in livers of chickens of different ages. Each data point represents the mean ± SD of 6 chickens (n = 6). The same letters are not significantly different, whereas different letters are significantly different. (**B**) Comparison between the expression curve of THRSP mRNA and the egg production curve of the Lushi green-shelled-egg chickens. Each point in the egg production curve represents the cumulative egg production of 2000 chickens in one week.

The expression levels of *THRSP* mRNAs in the livers of chickens at different developmental stages were detected by qPCR. The results showed that the expression levels of *THRSP* mRNA increased with sex maturation and reached the highest level at 30 weeks (Fig. 2A). According to egg production records, Lushi green-shelled-egg chickens start their first egg at an average age of 21 weeks old, and reach the peak rate of egg production at about 28 weeks. We further compared the expression pattern of the *THRSP* mRNA and the egg production curve of the Lushi green-shelled-egg chickens. Our data suggest a similar changing tend between the *THRSP* mRNA expression and the egg production (Fig. 2B).

### Effect of 17β-estradiol on *THRSP* expression in the liver and abdominal fat

**Figure 3:**
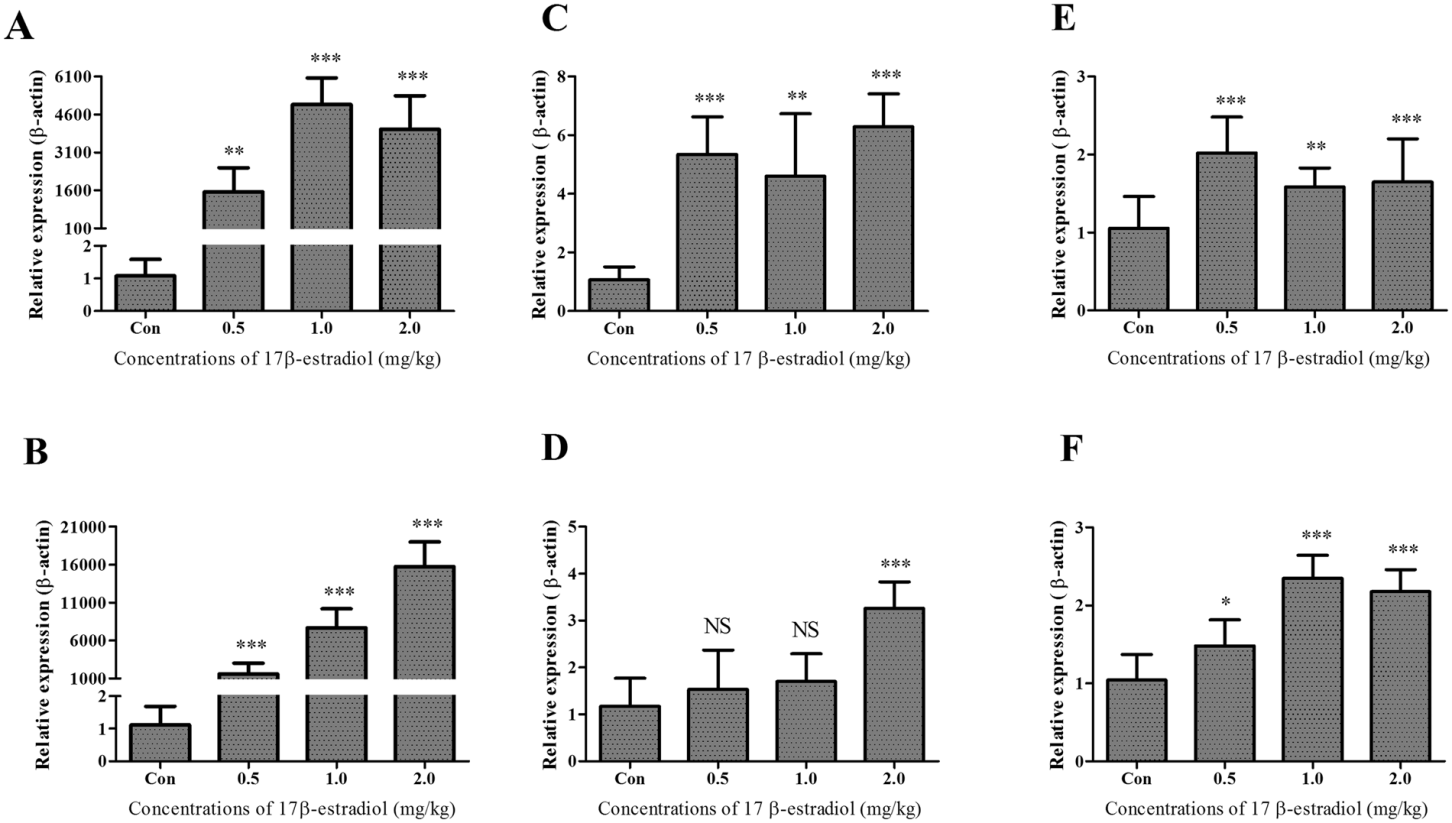
Effect of 17β-estradiol on *APOV1* and *THRSP* mRNA expressions in the liver and abdominal fat of chicken. (**A** and **B**) *APOV1* mRNA expression levels in chicken liver quantified by qPCR after different concentrations of estrogen treatment for 12 and 24 hours, respectively. (**C** and **D**) *THRSP* mRNA expression levels in chicken liver quantified by qPCR after different concentrations of estrogen treatment for 12 and 24 hours, respectively. (**E** and **F**) *THRSP* mRNA expression levels in chicken abdominal fat quantified by qPCR after different concentrations of estrogen treatment for 12 and 24 hours, respectively. Each data point represents the mean ± SD of 6 chickens (n = 6). *P < 0.05; **P < 0.01; ***P < 0.001; NS, no significant difference versus control.

**Figure 4:**
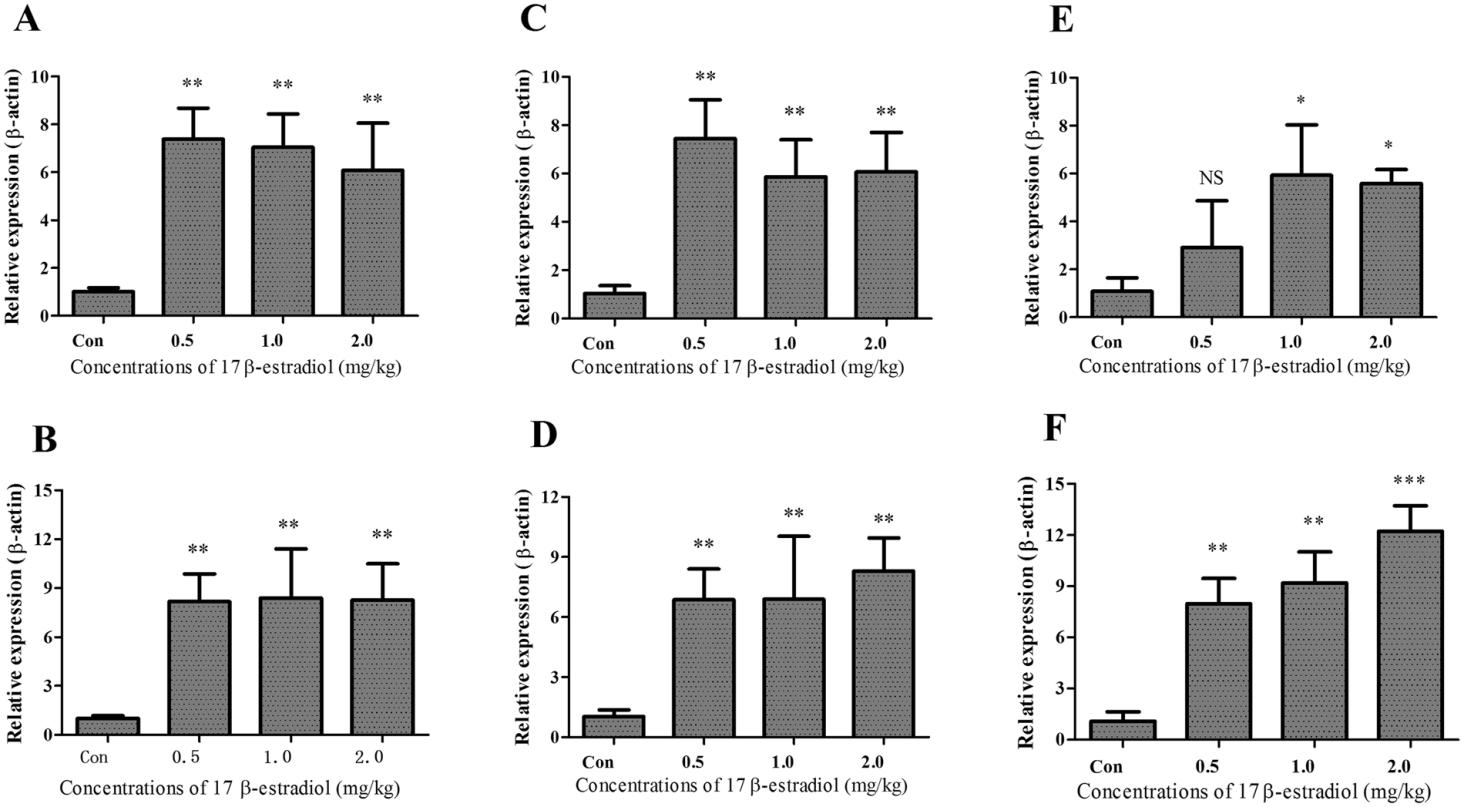
Effect of 17β-estradiol on *AACS*, *FASN* and *SCD* mRNA expressions in the liver of chickens. (**A**,**C** and **E**) *AACS*, *FASN* and *SCD* mRNA expression levels in chicken livers quantified by qPCR after they were treated with different doses of 17β-estradiol for 12 hours, respectively. (**B**,**D** and **E**) *AACS*, *FASN* and *SCD* mRNA expression levels in chicken livers quantified by qPCR after they were treated with different doses of 17β-estradiol for 24 hours, respectively. Each data point represents the mean ± SD of 6 chickens (n = 6). *P < 0.05; **P < 0.01; ***P < 0.001; NS, no significant difference versus control.

The upregulation of genes involved in lipogenesis in the liver of chickens was mainly induced by estrogen because estrogen levels were increasing with sex maturation, reached peak levels before the first egg and stayed at relatively higher levels during the egg production period^28, 29^. Therefore, it was speculated that expression of *THRSP* in the liver of chickens was regulated by estrogen. To assess this, the 10-week-old juvenile hens were treated with different concentrations of 17β-estradiol for various periods. The *APOV1* mRNA appears in chicken liver within a few hours after estrogen administration^34^, and its expression is strictly dependent on estrogen^24^. As a positive control, the expression levels of *APOV1* mRNA increased dramatically after 17β-estradiol treatment for 12 hours and 24 hours in the liver (*P* < 0.01) (Fig. 3A and B). Meanwhile, the expression levels of *THRSP* mRNA also significantly increased in both the liver and adipose tissue after 17β-estradiol treatment for 12 hours or 24 hours (Fig. 3C–F). We also measured expression levels of three lipogenic genes including *AACS*, *SCD* and *FASN* in livers of birds treated with or without 17β-estradiol. All three genes were significantly upregulated in chicken livers by the estradiol treatment (Fig. 4).

### Effect of 17β-estradiol on *THRSP* expression in primary hepatocytes

**Figure 5:**
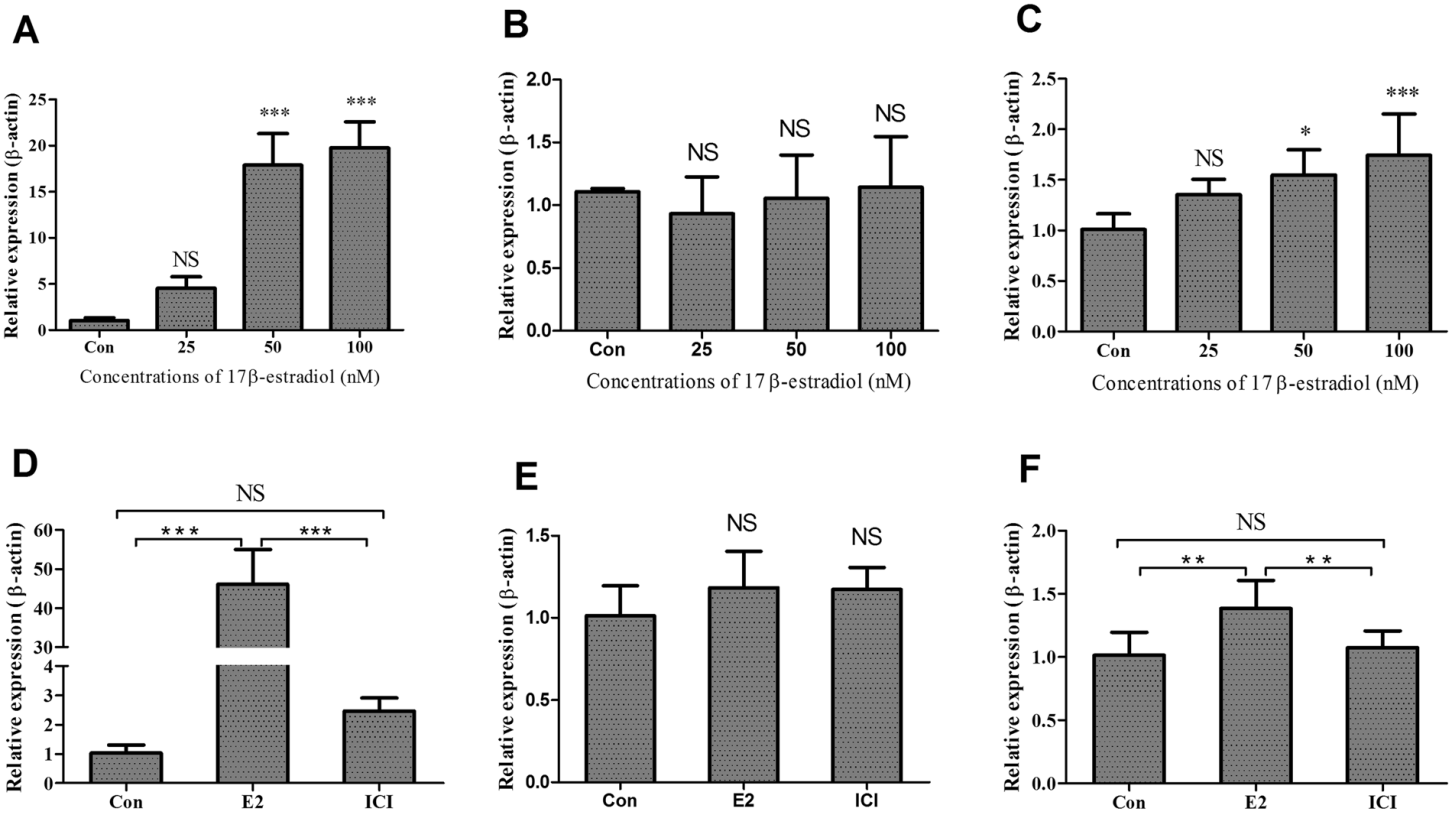
Effects of 17β-estradiol and ICI on *THRSP* expression in primary hepatocytes. (**A**,**B** and **C**) *APOV1*, *MC5R* and *THRSP* mRNA expression levels quantified by qPCR in primary hepatocytes treated by different doses of 17β-estradiol. (**D**,**E** and **F**) *APOV1*, *MC5R* and *THRSP* mRNA expression levels in primary hepatocytes treated by 100 nM 17β-estradiol and 10 nM ICI. Each data point represents the mean ± SD of 6 chickens (n = 6). *P < 0.05; **P < 0.01; ***P < 0.001; NS, no significant difference versus control.

To further verify whether the expression levels of THRSP were regulated by estrogen, *in vitro* cultured chicken embryonic primary hepatocytes were treated with different doses of 17β-estradiol. The expression levels of the *APOV1*, *MC5R* and *THRSP* genes were detected using qPCR. The expression levels of *APOV1* mRNA, as a positive control, were significantly upregulated by 17β-estradiol treatment (Fig. 5A). As previously demonstrated^35^, treatment with 17β-estradiol did not change the expression of the *MC5R* gene in chicken embryonic primary hepatocytes (Fig. 5B). The expression levels of *THRSP* mRNA increased in a dose-dependent manner (Fig. 5C).

To examine the effects of estrogen on the expression of *THRSP* gene further, the estrogen receptor antagonist ICI 182,780 together with 17β-estradiol were used to treat the *in vitro* cultured chicken embryonic primary hepatocytes. The results showed that the expression of *APOV1* and *THRSP* induced by 17β-estradiol was strongly antagonized by ICI 182,780 (Fig. 5D and F). However, the *MC5R* mRNA was not significantly changed by treatment with 17β-estradiol in the presence or absence of the ICI 182,780 (Fig. 5E).

### Effect of 17β-estradiol on thyroid hormone signaling in the liver

**Figure 6:**
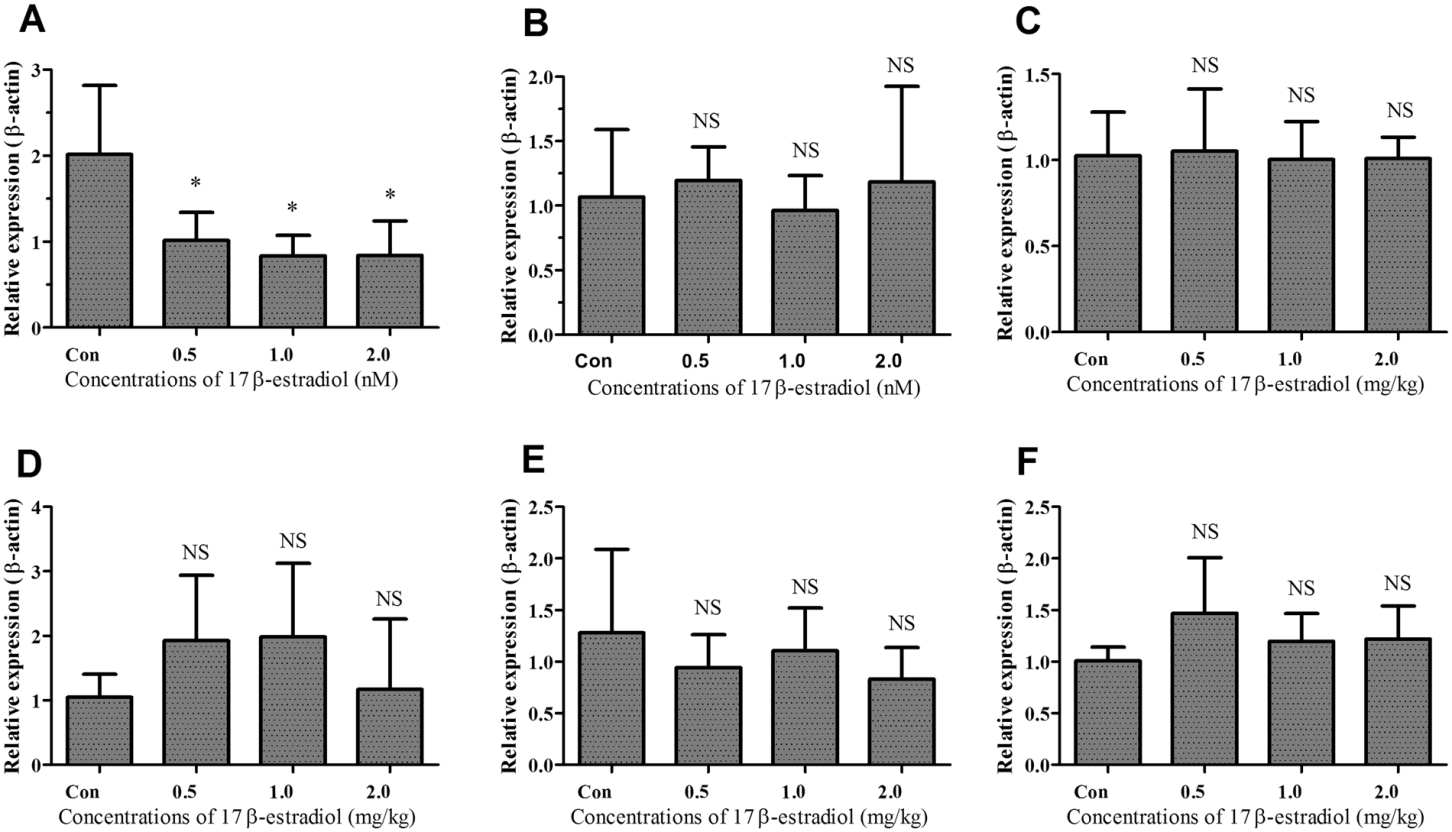
Effects of 17β-estradiol on TH relative genes expression levels in the liver. (**A**–**F**) *THRα*, *THRβ, Dio1*, *Dio2*, *ADH1B* and *PIK3CA* mRNA expression levels in chicken liver quantified by qPCR after they were treated with different doses of estrogen for 12 hours, respectively. Each data point represents the mean ± SD of 6 chickens (n = 6). *P < 0.05; **P < 0.01; NS, no significant difference versus control.

The *THRSP* gene can be rapidly upregulated by the thyroid hormone (TH)^1–4^, a component of the hypothalamic-pituitary-thyroid axis that is involved in the stress responses^38^. Previous studies demonstrated that the activity of thyroid hormones (THs) is regulated at the transcriptional level by specific nuclear receptors that are encoded by the TH receptor-α (THRα) and TH receptor-β (THRβ) genes^39, 40^. To determine whether the thyroid hormone signaling participates in the regulation of THRSP in the liver, we measured the mRNA expression levels of *THRα* and *THRβ*, and other known TH target genes including *Dio1*, *Dio2*, *ADH1B* and *PIK3CA* ^41, 42^ in the livers after treatment with 17β-estradiol. Although expression level of *THRα* was significantly downregulated in livers treated with 17β-estradiol compared to control (p < 0.05), no dramatic change of any other gene measured was observed (Fig. 6). These data indicate that the increase of THRSP by 17β-estradiol do not via TH singling pathway.

### Identification of the putative estrogen receptor binding site in the *THRSP* promoter

**Figure 7:**
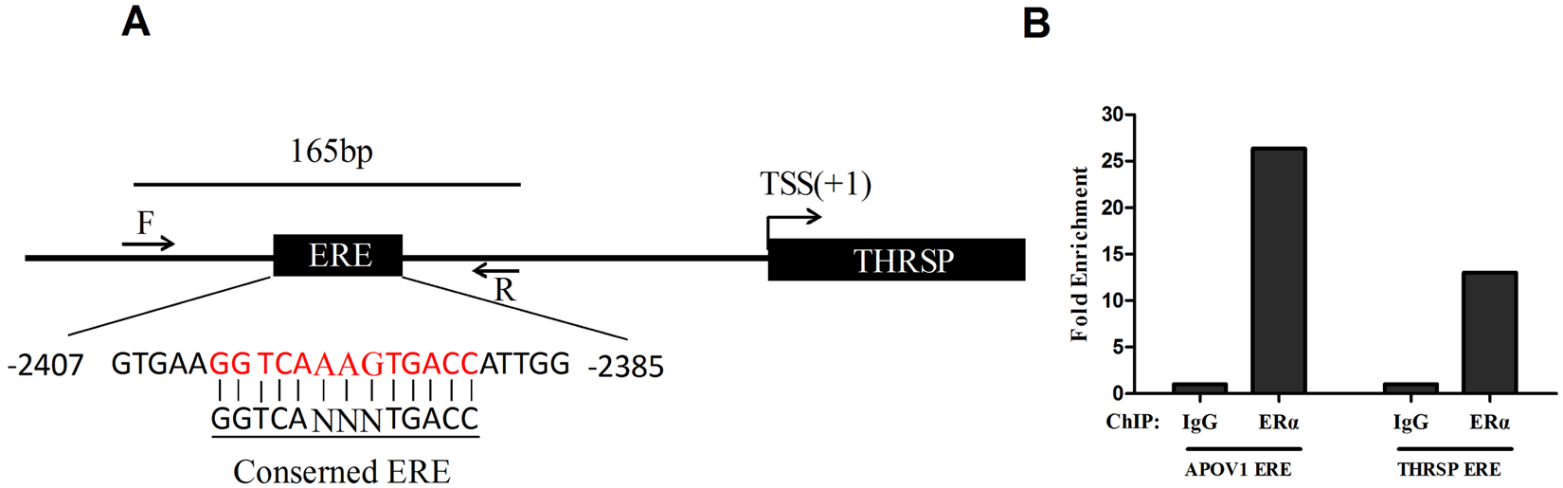
Identification of the putative estrogen receptor binding site in the *THRSP* promoter. (**A**) Location and sequence of the putative ERα binding site in the *THRSP* promoter. A conservative ERE was shown below the putative ERE; N represents any base. Arrows indicate the position of primers designed for qPCR amplification. (**B**) The binding of ERα to putative ERE regions in the THRSP promoter were analyzed by ChIP assays. After immunoprecipitation, ERE regions were amplified using qPCR. Pre-immune IgG was used as a negative control.

By sequence comparison analysis, a putative estrogen receptor binding site located between −2390 and −2402 bp upstream of the *THRSP* promoter was found (Fig. 7A). The ChIP-qPCR analysis demonstrated that the putative ERα binding site in the *THRSP* promoter sequence could be detected efficiently both in Input and IP samples as it was done in the positive control of the *APOV1* promoter binding site, where the gene IgG was a negative control. The fold enrichment was calculated and shown in Fig. 7B.

## Discussion

Many studies have reported that *THRSP* is expressed predominately in lipogenic tissues such as liver, fat and the mammary gland in mammalian systems^2, 3^. *THRSP* in birds was first identified as an EST in the liver of chickens, and its expression and regulation were not well understood. Here we performed analysis of the tissue distribution of *THRSP* mRNA both in juvenile hens and laying hens. Results were similar to mammalian systems in that *THRSP* had relatively high mRNA expression levels in lipogenic tissues, liver and abdominal fat (Fig. 1). A recent study on ducks compared the mRNA expression levels of *THRSP* in the liver, sebum cutaneum fat and abdominal fat collected from 6-week-old ducks^16^, and found that *THRSP* had a higher mRNA expression level in fat tissue than in the liver. In our results, we also found higher mRNA expression levels in abdominal fat than in the liver in juvenile hens. However, *THRSP* mRNA levels were 2-fold higher in the live compared with adipose in laying hens. It is known that the liver synthesizes more than 90% of the lipids. In contrast, abdominal fat is the main storage organization. The *THRSP* gene has been shown to be a lipogenic gene, and mostly involved in the liver lipid synthesis biological process in birds. The increase of *THRSP* gene expression in the laying hens implied a more active *THRSP* in the livers of laying hens than that in the livers of 10-week-old pullets. In this case, we detected the expression patterns of *THRSP* in the liver at different developmental stages; expression patterns were similar with the chicken egg production curve. Together, these results suggest that *THRSP* is involved in lipid metabolism in the liver of chickens.

*THRSP* plays an important role in lipogenic tissues and in regulating the key genes of lipogenesis^6–8^. Here we reported different *THRSP* mRNA expression levels between natural populations of juvenile and laying hens. The difference is easily associated with different physiologically active adipose synthesis in the livers of laying hens. It is reported that egg production behavior is controlled by the hypothalamic-pituitary-gonadal axis (HPG) and sex hormones, especially estrogen. The levels of estrogen in laying hens are significantly higher than in pullets^28, 29^. Estrogens initiate the transcription of estrogen-dependent genes and enhance the stability of the transcripts^43^. Our *in vivo* and *in vitro* studies strongly suggest that estrogen upregulates the expression level of *THRSP* (Figs 3 and 5). Meanwhile, our data also excluded the possibility that changes of expression level of *THRSP* in chicken liver treated with estrogen were caused by the TH singling pathway (Fig. 6). Therefore, we believe that *THRSP* is involved in the estrogen regulation network. We also observed that the expression of *THRα* was downregulated by estradiol treatment (Fig. 6A). It has been reported that there is a striking similarity between the ERE and TRE, resulting a repression of activated ERs by TRs^44, 45^. A recent study suggests that increased expression of THRSP is associated with ER-positive status in mouse with breast cancer^46^. It seems that the downregulation of THRα might be caused by the competitive inhibition or feedback regulation of the activated ERs. Importantly, the downregulation of THRα did not lead to the activation/inhibition of the TH singling pathway.

Another study reported that progesterone significantly increased the expression level of THRSP in female subcutaneous WAT in rat^47^. Considering the similarity of tissue distribution patterns of THRSP, we suggest that THRSP can be upregulated by estrogen in both between the chicken and the mammals.

To date, there are many transcription factor responsive elements in the promoter of *THRSP* in mammals, such as TRE, ChoRE, and SRE. Thus, the *THRSP* gene can be quickly activated by T3 and carbohydrate^4, 5^. To understand further the regulation mechanism of *THRSP* in chickens, we analyzed the 3000 bp promoter of *THRSP* bases to the transcriptional start site. In our data, we found a conscious ERE in the −2434 – −2422 range (Fig. 7A). Next we found that the ERE site is a positive binding site for ERα by ChIP-qPCR. We therefore conclude that *THRSP* is directly regulated by estrogen and is involved in the estrogen regulation network in chicken. Nevertheless, a perturbation of the ERE by gene editing or site-directed mutagenesis may further support this conclusion.

**Figure 8:**
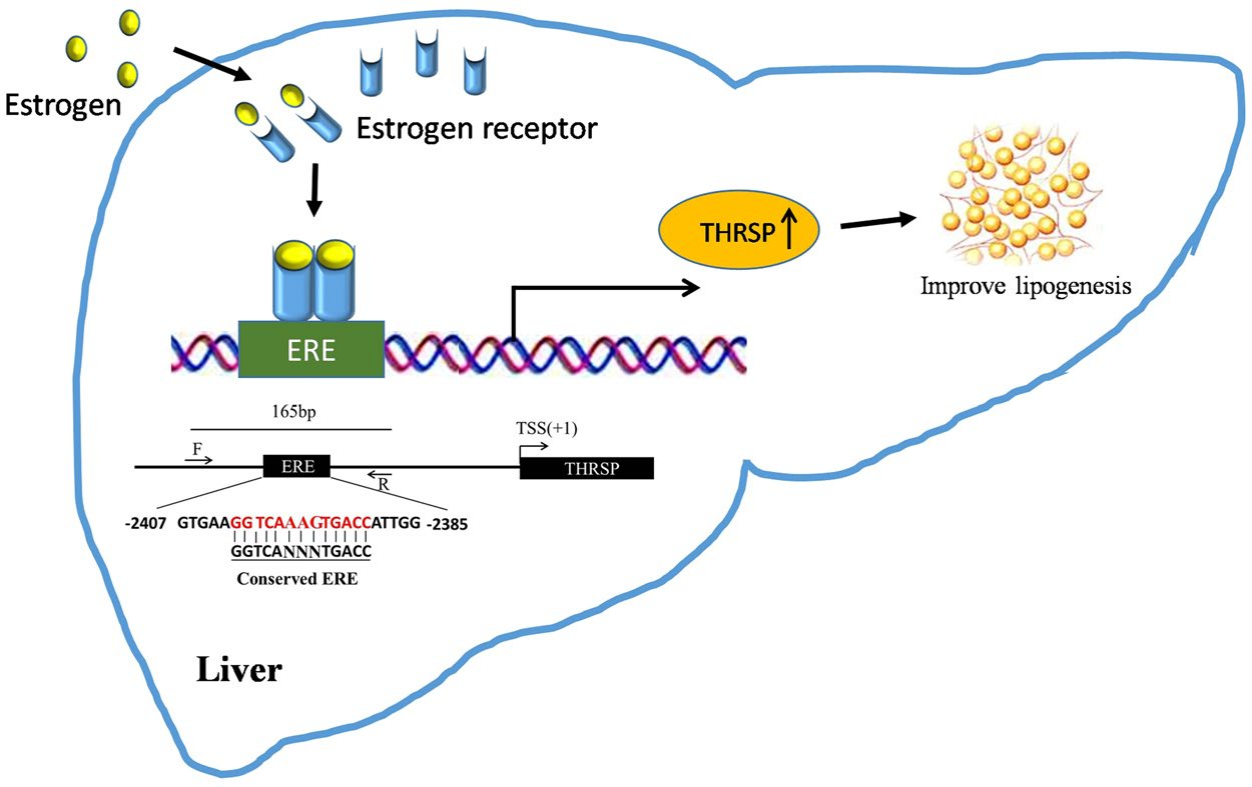
Model of estrogen-mediated regulation of THRSP.

*THRSP* has served as an important lipogenic gene, but the exact mechanism of *THRSP* involved in mammalian and avian lipid metabolism has not been well characterized. Here our study suggests that the chicken *THRSP* gene can be directly regulated by estrogen, which is considered to be the primary regulator of lipid metabolism (Fig. 8). This opens a new perspective in avian lipid metabolism. However, the synergistic and/or competitive actions of TH, estrogen, glucose, carbohydrate and other nutritional factors in *THRSP* expression need to be investigated further.

## References

[1] Seelig S, Liaw CW, Towle HC, Oppenheimer JH (1981). Thyroid hormone attenuates and augments hepatic gene expression at a pretranslational level. Proceedings of the National Academy of Sciences of the United States of America.

[2] Liaw CW, Towle HC (1984). Characterization of a thyroid hormone-responsive gene from rat. Journal of Biological Chemistry.

[3] Jump DB, Oppenheimer JH (1985). High basal expression and 3,5,3’-triiodothyronine regulation of messenger ribonucleic acid S14 in lipogenic tissues. Endocrinology.

[4] Tsatsos NG, Augustin LB, Anderson GW, Towle HC, Mariash CN (2008). Hepatic expression of the SPOT 14 (S14) paralog S14-related (Mid1 interacting protein) is regulated by dietary carbohydrate. Endocrinology.

[5] Jump DB, Clarke SD, Macdougald O, Thelen A (1993). Polyunsaturated fatty acids inhibit S14 gene transcription in rat liver and cultured hepatocytes. Proceedings of the National Academy of Sciences of the United States of America.

[6] Towle HC, Kaytor EN, Shih HM (2003). Regulation of the expression of lipogenic enzyme genes by carbohydrate. Annual Review of Nutrition.

[7] Aipoalani DL, O’Callaghan BL, Mashek DG, Mariash CN, Towle HC (2010). Overlapping roles of the glucose-responsive genes, S14 and S14R, in hepatic lipogenesis. Endocrinology.

[8] Kinlaw WB, Church JL, Harmon J, Mariash CN (1995). Direct evidence for a role of the “spot 14” protein in the regulation of lipid synthesis. Journal of Biological Chemistry.

[9] Kinlaw WB, Tron P, Witters LA (1993). Thyroid hormone and dietary carbohydrate induce different hepatic zonation of both “spot 14” and acetyl-coenzyme-A carboxylase: a novel mechanism of coregulation. Endocrinology..

[10] Brown SB, Maloney M, Kinlaw WB (1997). “Spot 14” Protein Functions at the Pretranslational Level in the Regulation of Hepatic Metabolism by Thyroid Hormone and Glucose. Journal of Biological Chemistry.

[11] Cui Y (2015). Thyroid hormone responsive protein spot 14 enhances lipogenesis in bovine mammary epithelial cells. In Vitro Cellular & Developmental Biology - Animal.

[12] Cogburn LA (2003). Systems-wide chicken DNA microarrays, gene expression profiling, and discovery of functional genes. Poult Sci.

[13] Ikeobi CON (2002). Quantitative trait loci affecting fatness in the chicken. Animal Genetics.

[14] Wang X, Carre W, Zhou H, Lamont SJ, Cogburn LA (2004). Duplicated Spot 14 genes in the chicken: characterization and identification of polymorphisms associated with abdominal fat traits. Gene..

[15] Grillasca JP (1997). Cloning and initial characterization of human and mouse Spot 14 genes. Febs Letters.

[16] Zhan K (2006). Molecular Cloning and Expression of the Duplicated Thyroid Hormone Responsive Spot 14 (THRSP) Genes in Ducks. Poultry Science.

[17] Campbell MC, Anderson GW, Mariash CN (2003). Human spot 14 glucose and thyroid hormone response: characterization and thyroid hormone response element identification. Endocrinology.

[18] Liu HC, Towle HC (1994). Functional synergism between multiple thyroid hormone response elements regulates hepatic expression of the rat S14 gene. Molecular Endocrinology.

[19] Mater MK, Thelen AP, Pan DA, Jump DB (1999). Sterol Response Element-binding Protein 1c (SREBP1c) Is Involved in the Polyunsaturated Fatty Acid Suppression of Hepatic S14 Gene Transcription. Journal of Biological Chemistry.

[20] Martel PM (2006). S14 protein in breast cancer cells: Direct evidence of regulation by SREBP-1c, superinduction with progestin, and effects on cell growth. Experimental Cell Research.

[21] Kumar V, Chambon P (1988). The estrogen receptor binds tightly to ITS responsive element as a ligand-induced homodimer. Cell.

[22] Paech K (1997). Differential ligand activation of estrogen receptors ERα and ERβ at AP1 sites. Science.

[23] Walker P, Germond JE, Brownluedi M, Givel F, Wahli W (1984). Sequence homologies in the region preceding the transcription initiation site of the liver estrogen-responsive vitellogenin and *APOV1* genes. Nucleic Acids Research.

[24] Kleinhitpass L, Ryffel GU, Heitlinger E, Cato AC (1988). A 13 bp palindrome is a functional estrogen responsive element and interacts specifically with estrogen receptor. Nucleic acids research.

[25] Hess DRA (2011). Estrogen and Its Receptors in Efferent Ductules and Epididymis. Journal of Andrology.

[26] Sato M, Rippy MK, Bryant HU (1996). Raloxifene, tamoxifen, nafoxidine, or estrogen effects on reproductive and nonreproductive tissues in ovariectomized rats. Faseb Journal.

[27] Li H (2015). Transcriptome profile of liver at different physiological stages reveals potential mode for lipid metabolism in laying hens. BMC Genomics.

[28] Tanabe Y, Nakamura T, Tanase H, Doi O (1981). Comparisons of plasma LH, progesterone, testosterone and estradiol concentrations in male and female chickens (Gallus domesticus) from 28 to 1141 days of age. Endocrinologia Japonica.

[29] Williams J, Harvey S (1986). Plasma concentrations of luteinizing hormone growth hormone, oestradiol, testosterone and androstenedione in the domestic hen from 6 weeks of age to sexual maturity. Reproduction Nutrition Development.

[30] Schneider, W. J. Chapter 17 – Lipoprotein Receptors. *Biochemistry of Lipids Lipoproteins & Membranes*, 489–518 (2016).

[31] Capony F, Williams DL (1980). Apolipoprotein B of avian very low density lipoprotein: characteristics of its regulation in nonstimulated and estrogen-stimulated rooster. Biochemistry.

[32] Wiskocil R (1980). Coordinate regulation of two estrogen-dependent genes in avian liver. Proceedings of the National Academy of Sciences of the United States of America.

[33] Gordon DA, Shelness GS, Nicosia M, Williams DL (1988). Estrogen-induced destabilization of yolk precursor protein mRNAs in avian liver. Journal of Biological Chemistry.

[34] Van HSF, Strijker R, Samallo J, Gruber M, Geert AB (1986). Conserved sequence motifs upstream from the co-ordinately expressed vitellogenin and *APOV1* genes of chicken. Nucleic Acids Research.

[35] Ren J (2016). Association of Estradiol on Expression of Melanocortin Receptors and Their Accessory Proteins in the Liver of Chicken (Gallus gallus). General & Comparative Endocrinology.

[36] Fischer PWF, Marks GS (1976). Chick embryo liver cells maintained in serum-free waymouth MD 705/1 medium. Methods in Cell Science.

[37] Nolan T, Hands RE, Bustin SA (2006). Quantification of mRNA using real-time RT-PCR. Nature Protocols.

[38] Helmreich DL, Parfitt DB, Lu XY, Akil H, Watson SJ (2005). Relation between the hypothalamic-pituitary-thyroid (HPT) axis and the hypothalamic-pituitary-adrenal (HPA) axis during repeated stress. Neuroendocrinology.

[39] Breuker C (2010). Hepatic Expression of Thyroid Hormone-Responsive Spot 14 Protein Is Regulated by Constitutive Androstane Receptor (NR1I3). Endocrinology.

[40] Zilz ND, Murray MB, Towle HC (1990). Identification of multiple thyroid hormone response elements located far upstream from the rat S14 promoter. Journal of Biological Chemistry.

[41] Moeller LC, Cao X, Dumitrescu AM (2006). Thyroid hormone mediated changes in gene expression can be initiated by cytosolic action of the thyroid hormone receptor beta through the phosphatidylinositol 3-kinase pathway. Nuclear Receptor Signaling.

[42] Moeller LC, Dumitrescu AM, Walker RL (2005). Thyroid hormone responsive genes in cultured human fibroblasts. Journal of Clinical Endocrinology & Metabolism.

[43] Flouriot G, Pakdel F, Valotaire Y (1996). Transcriptional and post-transcriptional regulation of rainbow trout estrogen receptor and vitellogenin gene expression. Molecular & Cellular Endocrinology.

[44] Graupner G, Zhang XK, Tzukerman M (1991). Thyroid hormone receptors repress estrogen receptor activation of a TRE. Molecular Endocrinology.

[45] Glass CK, Holloway JM, Devary OV (1988). The thyroid hormone receptor binds with opposite transcriptional effects to a common sequence motif in thyroid hormone and estrogen response elements. Cell.

[46] Anderson SM (2013). Elevated THRSP/Spot14 expression is correlated with improved clinical outcome in breast cancer which is consistent with reduced metastasis and enhanced differentiation observed in the MMTV-Neu mouse model. Cancer Epidemiology Biomarkers & Prevention.

[47] Stelmanska E, Swierczynski J (2013). Up-regulation of lipogenic enzyme genes expression in inguinal white adipose tissue of female rats by progesterone. Journal of Steroid Biochemistry & Molecular Biology.

